# Numerical simulation of natural convection in a square enclosure filled with nanofluid using the two-phase Lattice Boltzmann method

**DOI:** 10.1186/1556-276X-8-56

**Published:** 2013-02-04

**Authors:** Cong Qi, Yurong He, Shengnan Yan, Fenglin Tian, Yanwei Hu

**Affiliations:** 1 School of Energy Science and Engineering, Harbin Institute of Technology, Harbin, 150001, China

**Keywords:** Two phase Lattice Boltzmann model, Rayleigh number, Nanofluid, Natural convection

## Abstract

Considering interaction forces (gravity and buoyancy force, drag force, interaction potential force, and Brownian force) between nanoparticles and a base fluid, a two-phase Lattice Boltzmann model for natural convection of nanofluid is developed in this work. It is applied to investigate the natural convection in a square enclosure (the left wall is kept at a high constant temperature (*T*_H_), and the top wall is kept at a low constant temperature (*T*_C_)) filled with Al_2_O_3_/H_2_O nanofluid. This model is validated by comparing numerical results with published results, and a satisfactory agreement is shown between them. The effects of different nanoparticle fractions and Rayleigh numbers on natural convection heat transfer of nanofluid are investigated. It is found that the average Nusselt number of the enclosure increases with increasing nanoparticle volume fraction and increases more rapidly at a high Rayleigh number. Also, the effects of forces on nanoparticle volume fraction distribution in the square enclosure are studied in this paper. It is found that the driving force of the temperature difference has the biggest effect on nanoparticle volume fraction distribution. In addition, the effects of interaction forces on flow and heat transfer are investigated. It is found that Brownian force, interaction potential force, and gravity-buoyancy force have positive effects on the enhancement of natural convective heat transfer, while drag force has a negative effect.

## Background

Compared with common fluids such as water, nanofluid, using nanoscale particles dispersed in a base fluid, has an effect of enhancing the performance of natural convection heat transfer due to its high heat conductivity coefficient. Many researchers investigated nanoparticles and nanofluid in recent years. Wang et al. [1] synthesized stimuli-responsive magnetic nanoparticles and investigated the effect of nanoparticle fraction on its cleavage efficiency. Bora and Deb [2] developed a novel bioconjugate of stearic acid-capped maghemite nanoparticle (γ-Fe_2_O_3_) with bovine serum albumin. Guo et al. [3] produced magnetic nanofluids containing γ-Fe_2_O_3_ nanoparticles using a two-step method, measured their thermal conductivities and viscosity, and tested their convective heat transfer coefficients. Pinilla et al. [4] investigated the growth of Cu nanoparticles in a plasma-enhanced sputtering gas aggregation-type growth region. Yang and Liu [5] produced a kind of stable nanofluid by surface functionalization of silica nanoparticles. Zhu et al. [6] developed a wet chemical method to produce stable CuO nanofluids. Nadeem and Lee [7] investigated the steady boundary layer flow of nanofluid over an exponential stretching surface. Wang and Fan [8] reviewed the nanofluid research in the last 10 years.

Natural convection is applied in many fields, and extensive researches have been performed. Oztop et al. [9] and Ho et al. [10] respectively investigated natural convection in partially heated rectangular enclosures and discussed the effects of viscosity and thermal conductivity of nanofluid on laminar natural convection heat transfer in a square enclosure by a finite-volume method. Saleh et al. [11] investigated heat transfer enhancement utilizing nanofluids in a trapezoidal enclosure by a finite difference approach. Ghasemi et al. [12], Santra et al. [13], and Aminossadati et al. [14] numerically simulated natural convection in a triangular enclosure and studied the behavior of natural convection heat transfer in a differentially heated square cavity, described a study on natural convection of a heat source embedded in the bottom wall of an enclosure, and used the SIMPLE algorithm to solve the governing equation. Kargar et al. [15] used computational fluid dynamics and an artificial neural network to investigate the cooling performance of two electronic components in an enclosure. Abu-Nada et al. [16] investigated the effect of variable properties on natural convection in enclosures filled with nanofluid, and the governing equations are solved by an efficient finite-volume method. Hwang et al. [17] investigated the thermal characteristics of natural convection in a rectangular cavity heated from below by Jang and Choi's model [18].

The Lattice Boltzmann method is a new way to investigate natural convection. Compared with the above traditional methods, the Lattice Boltzmann method has many merits including that boundary conditions can be conveniently dealt with, the transform between macroscopic and microscopic equations is easily achieved, the details of the fluid can be presented, and so on. In addition, nanofluid as the media can enhance heat transfer due to factors such as nanofluids having higher thermal conductivity and the nanoparticles in the fluid disturbing the laminar flow. Therefore, many researchers undertook investigations on the natural convection of nanofluids by the Lattice Boltzmann method. Barrios et al. [19] developed a Lattice Boltzmann model and applied it to investigate the natural convection of an enclosure with a partially heated left wall. Peng et al. [20] presented a simple a Lattice Boltzmann model without considering thermal diffusion, and this model is easily applied because it does not contain a gradient term. He et al. [21] proposed a new Lattice Boltzmann model which introduced an internal energy distribution function to simulate the temperature field, and the result has a good agreement with the benchmark solution. Nemati et al. [22] simulated the natural convection of a lid-driven flow filled with Cu-water, CuO-water, and Al_2_O_3_-water nanofluids and discussed the effects of nanoparticle volume fraction and Reynolds number on the heat transfer. Wang et al. [23] presented a Lattice Boltzmann algorithm to simulate the heat transfer of a fluid-solid fluid, and the result has a satisfactory agreement with the published data. Dixit et al. [24] applied the Lattice Boltzmann method to investigate the natural convection of a square cavity at high Rayleigh numbers. Peng et al. [25] developed a 3D incompressible thermal Lattice Boltzmann model for natural convection in a cubic cavity. The above Lattice Boltzmann methods are all single-phase models, and the nanofluid was seen as a single-phase fluid without considering the interaction forces between nanoparticles and water. In addition, the effects of these interaction forces on heat transfer were disregarded.

There are few two-phase lattice Boltzmann models that consider the interaction forces between nanoparticles and a base fluid for natural convection in an enclosure. Xuan et al. [26] proposed a two-phase Lattice Boltzmann model to investigate sudden-start Couette flow and convection in parallel plate channels without researching the effect of forces on volume fraction distribution of nanoparticles. Because these forces were not investigated before our work, the effects of forces between water and nanoparticles on the fluid flow patterns were unknown. In addition, as we know, the nanoparticles in the fluid easily gather together and deposit, especially at high volume fraction. Hence, the nanoparticle distribution in the fluid flow is important for nanofluid application, which is another objective in our paper. However, the single-phase model cannot be used to investigate nanoparticle distribution. Furthermore, natural convection of a square enclosure (left wall kept at a high constant temperature (*T*_H_), and top wall kept at a low constant temperature (*T*_C_)) filled with nanofluid is not investigated in the published literatures. In this paper, a two-phase Lattice Boltzmann model is proposed and applied to investigate the natural convection of a square enclosure (left wall kept at a high constant temperature (*T*_H_), and top wall kept at a low constant temperature (*T*_C_)) filled with Al_2_O_3_-water nanofluid and the inhomogeneous distribution of nanoparticles in the square enclosure.

## Methods

### Lattice Boltzmann method

The density distribution function for a single-phase fluid is calculated as follows:

(1) $$\begin{array}{ll} f_{\alpha }^{\sigma } & \left(r+e_{\alpha }\delta_{t},t+\delta_{t}\right)-f_{\alpha }^{\sigma }\left(r,t\right)\\ & =-\frac{1}{\tau_{f}^{\sigma }}\left[f_{\alpha }^{\sigma }\left(r,t\right)-f_{\alpha }^{\mathrm{\sigma eq}}\left(r,t\right)\right]+\delta_{t}F_{\alpha }^{\sigma '} \end{array}$$

(2) $$F_{\alpha }^{\sigma '}=G\cdot \frac{\left(e_{\alpha }-u^{\sigma }\right)}{p}f_{\alpha }^{\mathrm{\sigma eq}}$$

where $\tau_{f}^{\sigma }$ is the dimensionless collision-relaxation time for the flow field, ***e***_*α*_ is the lattice velocity vector, the subscript *α* represents the lattice velocity direction, $f_{\alpha }^{\sigma }\left(r,t\right)$ is the distribution function of the nanofluid with velocity ***e***_*α*_ (along the direction *α*) at lattice position ***r*** and time ***t***, $f_{\alpha }^{\mathrm{\sigma eq}}\left(r,t\right)$ is the local equilibrium distribution function, *δ*_*t*_ is the time step, *δ*_*x*_ is the lattice step, the order numbers *α* = 1,…,4 and *α* = 5,…,8, respectively represent the rectangular directions and the diagonal directions of the lattice, $F_{\alpha }^{\sigma '}$ is the external force term in the direction of the lattice velocity without interparticle interaction, *G* = - *β*(*T*_*nf*_ - *T*_0_)***g*** is the effective external force, where ***g*** is the gravity acceleration, *β* is the thermal expansion coefficient, *T*_*nf*_ is the temperature of the nanofluid, and *T*_0_ is the mean value of the high and low temperature of the walls.

A nanofluid is a two-phase fluid constituted by nanoparticles and a base fluid, and there are interaction forces (gravity and buoyancy force, drag force, interaction potential force, and Brownian force) between nanoparticles and the base fluid. Thus, the macroscopic density and velocity fields are simulated using the density distribution function by adding the forces term.

(3) $$\begin{array}{ll} f_{\alpha }^{\sigma } & \left(r+e_{\alpha }\delta_{t},t+\delta_{t}\right)-f_{\alpha }^{\sigma }\left(r,t\right)\\ & =-\frac{1}{\tau_{f}^{\sigma }}\left[f_{\alpha }^{\sigma }\left(r,t\right)-f_{\alpha }^{\mathrm{\sigma eq}}\left(r,t\right)\right]\\ & \;+\frac{2\tau_{f}^{\sigma }-1}{2\tau_{f}^{\sigma }}\cdot \frac{F_{\alpha }^{\sigma }\delta_{t}e_{\alpha }}{B_{\alpha }c^{2}}+\delta_{t}F_{\alpha }^{\sigma '} \end{array}$$

where $F_{\alpha }^{\sigma }$ represents the total interparticle interaction forces, and *B*_*α*_ is one of the weight coefficients. $\frac{2\tau_{f}^{\sigma }-1}{2\tau_{f}^{\sigma }}$ is a coefficient. Because the total interparticle interaction forces cannot be optionally added in the lattice Boltzmann equation, we introduce an unknown coefficient in the total interparticle interaction forces. In order to enable the lattice Boltzmann equation including the total interparticle interaction forces to recover to the Navier-Stokes equation, based on the mass and momentum conservation, we used multi-scale technique to deduce the unknown coefficient which is equal to $\frac{2\tau_{f}^{\sigma }-1}{2\tau_{f}^{\sigma }}$. Due to the very long derivation process, we directly gave the final result in the paper.

The weight coefficient *B*_*α*_ is given as:

(4) $$B_{\alpha }=\{\begin{array}{l} 0\;\alpha =0\\ \frac{1}{3}\;\alpha =1,\ldots ,4\\ \frac{1}{12}\;\alpha =5,\ldots ,8 \end{array}$$

For the two-dimensional nine-velocity LB model (D2Q9) considered herein, the discrete velocity set for each component *α* is:

(5) $$e_{\alpha }=\{\begin{array}{ll} \left(0,0\right) & \alpha =0\\ c\left(\cos\left[\left(\alpha -1\right)\frac{\pi }{2}\right],\sin\left[\left(\alpha -1\right)\frac{\pi }{2}\right]\right) & \alpha =1,2,3,4\\ \sqrt{2}c\left(\cos\left[\left(2\alpha -1\right)\frac{\pi }{4}\right],\sin\left[\left(2\alpha -1\right)\frac{\pi }{4}\right]\right) & \alpha =5,6,7,8 \end{array}$$

The density equilibrium distribution function is chosen as follows:

(6) $$f_{\alpha }^{\mathrm{\sigma eq}}=\rho^{\sigma }w_{\alpha }\left[1+\frac{e_{\alpha }\cdot u^{\sigma }}{c_{s}^{2}}+\frac{{\left(e_{\alpha }\cdot u^{\sigma }\right)}^{2}}{2c_{s}^{4}}-\frac{u^{\sigma }{}^{2}}{2c_{s}^{2}}\right]$$

(7) $$w_{\alpha }=\{\begin{array}{l} \frac{4}{9}\;\alpha =0\\ \frac{1}{9}\;\alpha =1,\ldots ,4\\ \frac{1}{36}\;\alpha =5,\ldots ,8 \end{array}$$

where $c_{s}^{2}=\frac{c^{2}}{3}$ is the lattice's sound velocity, and *w*_*α*_ is the weight coefficient.

The macroscopic temperature field is simulated using the temperature distribution function.

(8) $$\begin{array}{l} T_{\alpha }^{\sigma }\left(r+e_{\alpha }\delta_{t},t+\delta_{t}\right)-T_{\alpha }^{\sigma }\left(r,t\right)\\ \;=-\frac{1}{\tau_{T}^{\sigma }}\left[T_{\alpha }^{\sigma }\left(r,t\right)-T_{\alpha }^{\mathrm{\sigma eq}}\left(r,t\right)\right] \end{array}$$

where *τ*_*T*_ is the dimensionless collision-relaxation time for the temperature field.

The temperature equilibrium distribution function is chosen as follows:

(9) $$T_{\alpha }^{\mathrm{\sigma eq}}=w_{a}T^{\sigma }\left[1+3\frac{e_{\alpha }\cdot u^{\sigma }}{c^{2}}+4.5\frac{{\left(e_{\alpha }\cdot u^{\sigma }\right)}^{2}}{2c^{4}}-1.5\frac{u^{\sigma }{}^{2}}{2c^{2}}\right]$$

In the case of no internal forces and external forces, the macroscopic temperature, density and velocity are respectively calculated as follows:

(10) $$T^{\sigma }=\sum_{\alpha =0}^{8}T_{\alpha }^{\sigma }$$

(11) $$\rho^{\sigma }=\sum_{\alpha =0}^{8}f_{\alpha }^{\sigma }$$

(12) $$u^{\sigma }=\frac{1}{\rho^{\sigma }}\sum_{\alpha =0}^{8}f_{\alpha }^{\sigma }e_{\alpha }$$

Considering the internal and external forces, the macroscopic velocities for nanoparticles and base fluid are modified to:

(13) $$u_{\mathrm{pnew}}=u_{p}+\frac{F_{p}\mathrm{\Delta t}e_{\alpha }}{2\rho^{\sigma }}$$

(14) $$u_{\mathrm{wnew}}=u_{w}+\frac{\mathrm{\Delta t}F_{W}}{2L_{x}L_{y}\rho^{w}}$$

where ***F***_p_ represents the total forces acting on the nanoparticles, ***F***_w_ represents the total forces acting on the base fluid, and *L*_*x*_*L*_*y*_ represents the total number of lattices.

When the internal forces and external forces are considered, energy between nanoparticles and base fluid is exchanged, and the macroscopic temperature for nanoparticles and base fluid is then given as:

(15) $$T_{\mathrm{new}}^{\sigma }=T^{\sigma }+\delta_{t}\tau_{T}\frac{\mathrm{dT}}{\mathrm{dt}}=T^{\sigma }+\delta_{t}\tau_{T}\Phi_{\alpha \beta }$$

where *Φ*_*αβ*_ is the energy exchange between nanoparticles and base fluid, $\Phi_{\alpha \beta }=\frac{h_{\alpha \beta }\left[T_{\beta }\left(x,t-\delta_{t}\right)-T_{\alpha }\left(x,t-\delta_{t}\right)\right]}{\rho_{\alpha }c_{\mathrm{p\alpha}}a_{\alpha }}$, and *h*_*αβ*_ is the convective heat transfer coefficient of the nanofluid.

The corresponding kinematic viscosity and thermal diffusion coefficients are respectively defined as follows:

(16) $$\nu^{\sigma }=\frac{1}{3}c^{2}\left(\tau_{f}^{\sigma }-\frac{1}{2}\right)\delta_{t}$$

(17) $$\chi^{\sigma }=\frac{1}{3}c^{2}\left(\tau_{T}^{\sigma }-\frac{1}{2}\right)\delta_{t}$$

The dimensionless collision-relaxation times *τ*_*f*_ and *τ*_*T*_ are respectively given as follows:

(18) $$\tau_{f}^{\sigma }=0.5+\frac{\mathrm{Ma}H\sqrt{3\mathrm{Pr}}}{c^{2}\mathrm{\delta t}\sqrt{\mathrm{Ra}}}$$

(19) $$\tau_{T}^{\sigma }=0.5+\frac{3\nu }{\mathrm{Pr}c^{2}\mathrm{\delta t}}$$

where Ma = 0.1, *H* = 1, *c* = 1, *δt* = 1, and the other parameters equations are given as follows:

(20) $$\mathrm{Pr}=\frac{\nu }{\alpha }=\frac{c_{p}\mu }{k}$$

(21) $$\nu =\frac{\mu }{\rho }$$

From Equations 18 and 19, the collision-relaxation time for the flow field and the temperature field can be calculated. For water phase, the *τ*_*f*_ collision-relaxation times are respectively 0.51433 and 0.501433 at Ra = 10^3^ and Ra = 10^5^, and the collision-relaxation time *τ*_*T*_ is 0.5. For nanoparticle phase, the *τ*_*f*_ collision-relaxation times are respectively 0.50096 and 0.500096 at Ra = 10^3^ and Ra = 10^5^, and the collision-relaxation time *τ*_*T*_ is 0.500025.

### Interaction forces between base fluid and nanoparticles

As noted before, a nanofluid is, in reality, a kind of two-phase fluid. There are interaction forces between liquid and nanoparticles which affect the behavior of the nanofluid. The external forces include gravity and buoyancy forces ***F***_H_, and the interparticle interaction forces include drag force (Stokes force) ***F***_D_, interaction potential ***F***_A_, and Brownian force ***F***_B_. We introduce them as follows.

The gravity and buoyancy force is given as:

(22) $$F_{H}=-\frac{4\pi a^{3}}{3}\mathrm{g\Delta}\rho^{'}$$

where *a* is the radius of a nanoparticle, and *Δρ*^'^ is the mass density difference between the suspended nanoparticle and the base fluid.

The drag force (Stokes force) is given as:

(23) $$F_{D}=-6\mathrm{\pi \mu a\Delta u}$$

where *μ* is the viscosity of the fluid, and ∆*u* is the velocity difference between the nanoparticle and the base fluid.

The interaction potential is presented as [27]:

(24) $$V_{A}=-\frac{1}{6}A\left(\frac{2a^{2}}{L_{\mathrm{cc}}^{2}-4a^{2}}+\frac{2a^{2}}{L_{\mathrm{cc}}^{2}}+\frac{L_{\mathrm{cc}}^{2}-4a^{2}}{L_{\mathrm{cc}}^{2}}\right)$$

where *A* is the Hamaker constant, and *L*_*cc*_ is the center-to-center distance between particles.

The interaction potential force is shown as:

(25) $$F_{A}=\sum_{i=1}^{8}n_{i}\frac{\partial V_{A}}{\partial r_{i}}$$

where *n*_*i*_ is the number of the particles within the adjacent lattice *i*, *n*_*i*_ = *ρ*^*σ*^*V*/*m*^*σ*^, *m*^*σ*^ is the mass of a single nanoparticle, and *V* is the volume of a single lattice.

The Brownian force is calculated as [28]:

(26) $$F_{B}=G_{i}\sqrt{\frac{C}{\mathrm{dt}}}$$

where *G*_*i*_ is a Gaussian random number with zero mean and unit variance, which is obtained from a program written by us, and *C* = 2*γk*_*B*_*T* = 2 × (6*πηa*)*k*_*B*_*T*, *γ* is the surface tension, *k*_*B*_ is the Boltzmann constant, *T* is the absolute temperature, and *η* is the dynamic viscosity.

The total per unit volume forces acting on nanoparticles of a single lattice is:

(27) $$F_{p}=n\left(F_{H}+F_{D}+F_{A}+F_{B}\right)/V$$

where *n* is the number of the particles in the given lattice, and *V* is the lattice volume.

In a nanofluid, the forces acting on the base fluid are mainly drag force and Brownian force. Thus the force acting on the base fluid in a given lattice is:

(28) $$F_{w}=-n\left(F_{D}+F_{B}\right)$$

## Results and discussion

**Figure 1:**
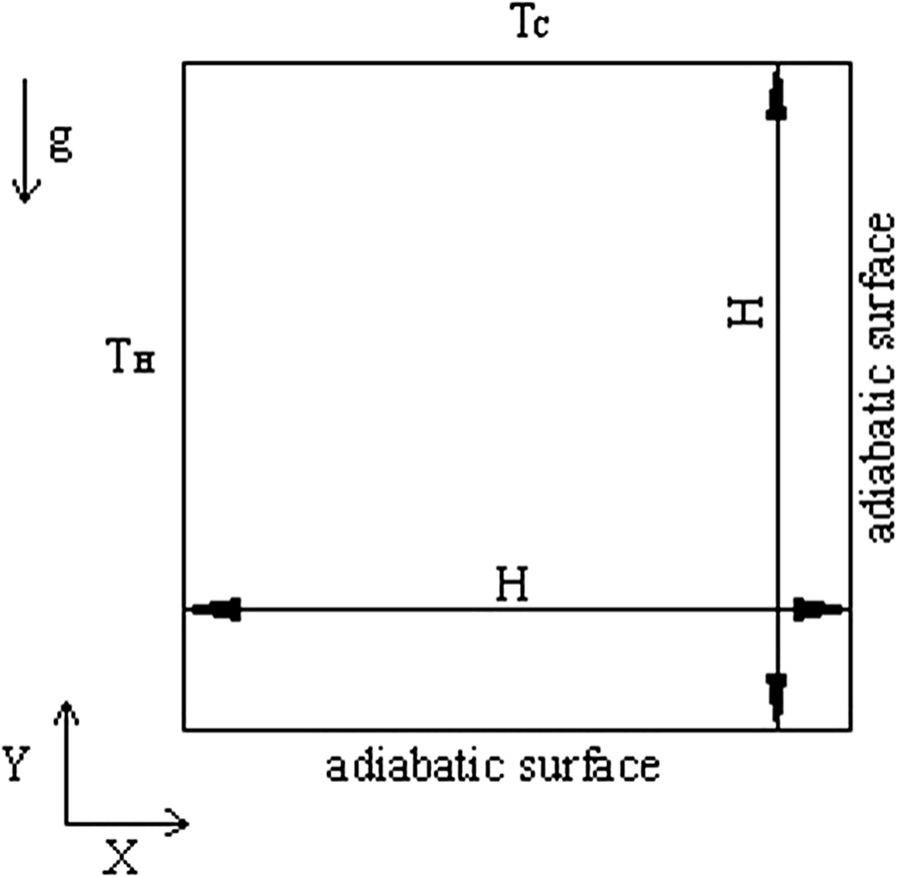
Schematic of the square cavity.

The two-phase Lattice Boltzmann model is applied to simulate the natural convection heat transfer in a square cavity which is shown in Figure 1. The square cavity is filled with the Al_2_O_3_-water nanofluid. The thermo-physical properties of water and Al_2_O_3_ are given in Table 1. The height and the width of the enclosure are both *H*. The left wall is kept at a high constant temperature (*T*_H_), and the top cold wall is kept at a low constant temperature (*T*_C_). The boundary conditions of the other walls (right wall and bottom wall) are all adiabatic. The initial conditions for the four walls are given as follows:

(29) $$\left\{ \begin{array}{llllll} x=0 & u=0, & T=1; & x=1 & u=0, & \partial T/\partial y=0\\ y=0 & u=0, & \partial T/\partial y=0; & y=1 & u=0, & T=0 \end{array}\right.$$

**Table 1:** **Thermo-physical properties of water and Al**_**2**_**O**_**3 **_[29]

**Physical properties**	**Fluid phase (H**_**2**_**O)**	**Nanoparticles (Al**_**2**_**O**_**3**_**)**
*ρ* (kg/m^3^)	997.1	3970
*c*_p_ (J/kg k)	4179	765
*v* (m^2^/s)	0.001004	-
*k* (W/m/K)	0.613	25

In the simulation, a non-equilibrium extrapolation scheme is adopted to deal with the boundary, and the criteria of the program convergence for the flow field and the temperature field are respectively given as follows:

(30) $${\text{Error}}_{1}=\frac{\sqrt{\sum_{i,j}\left\{{\left[u_{x}^{\sigma }\left(i,j,t+\delta_{t}\right)-u_{x}^{\sigma }\left(i,j,t\right)\right]}^{2}+{\left[u_{y}^{\sigma }\left(i,j,t+\delta_{t}\right)-u_{y}^{\sigma }\left(i,j,t\right)\right]}^{2}\right\}}}{\sqrt{\sum_{i,j}\left[u_{x}^{\sigma }{\left(i,j,t+\delta_{t}\right)}^{2}+u_{y}^{\sigma }{\left(i,j,t+\delta_{t}\right)}^{2}\right]}}<\varepsilon_{1}$$

(31) $${\mathrm{Error}}_{2}=\frac{\sqrt{\sum_{i,j}{\left[T^{\sigma }\left(i,j,t+\delta_{t}\right)-T^{\sigma }\left(i,j,t\right)\right]}^{2}}}{\sqrt{\sum_{i,j}T^{\sigma }{\left(i,j,t+\delta_{t}\right)}^{2}}}<\varepsilon_{2}$$

where *ε* is a small number, for example, for Ra = 1 × 10^3^, *ε*_1_ = 10^-6^, and *ε*_2_ = 10^-6^. About 2 weeks is needed to achieve the equilibrium state for the low Rayleigh number (Ra = 1 × 10^3^), and about 1 month for the high Rayleigh number (Ra = 1 × 10^5^).

The Nusselt number can be expressed as:

(32) $$\mathrm{Nu}=\frac{\mathrm{hH}}{k_{\mathrm{nf}}}\text{.}$$

The heat transfer coefficient is computed from:

(33) $$h=\frac{q_{w}}{T_{H}-T_{L}}\text{.}$$

The thermal conductivity of the nanofluid is defined by:

(34) $$k_{\mathrm{nf}}=-\frac{q_{w}}{\partial T/\partial x}\text{.}$$

Substituting Equations 33 and 34 into Equation 32, the local Nusselt number along the left wall can be written as:

(35) $$\mathrm{Nu}=-\left(\frac{\partial T}{\partial x}\right)\cdot \frac{H}{T_{H}-T_{L}}\text{.}$$

The average Nusselt number is determined from:

(36) $${\mathrm{Nu}}_{\mathrm{avg}}=\int_{0}^{1}\mathrm{Nu}\left(y\right)\mathrm{dy}\text{.}$$

In order to perform a grid independence test and validate the Lattice Boltzmann model proposed in this work, we used another square enclosure, because there are exact solutions for this square enclosure. The left wall is kept at a high constant temperature (*T*_H_), and the right wall is kept at a low constant temperature (*T*_C_). The boundary conditions of the other walls (top wall and bottom wall) are all adiabatic, and the other conditions are the same as those in Figure 1.

As shown in Table 2, the grid independence test is performed in a square enclosure using successively sized grids, 128 × 128, 192 × 192, 256 × 256, and 320 × 320 at Ra = 1 × 10^5^, Pr = 0.7. It can be seen from Table 2 that there is a bigger difference between the result obtained with grid sizes 128 × 128 and 192 × 192 and the result available from the literature [30] than when compared with the result obtained with grids 256 × 256 and 320 × 320. In addition, the result with grid 256 × 256 and the result with grid 320 × 320 are very close. In order to accelerate the numerical simulation, a grid size of 256 × 256 was chosen as a suitable one which can guarantee a grid-independent solution.

**Table 2:** **Comparison of the mean Nusselt numbers with different grids (*Ra *= 1 × 10**^**5**^**, *Pr *= 0.7)**

**Physical properties**	**128 × 128**	**192 × 192**	**256 × 256**	**320 × 320**	**Literature**[30]
*Nu*_avg_	4.5466	4.5251	4.5220	4.5218	4.5216

**Figure 2:**
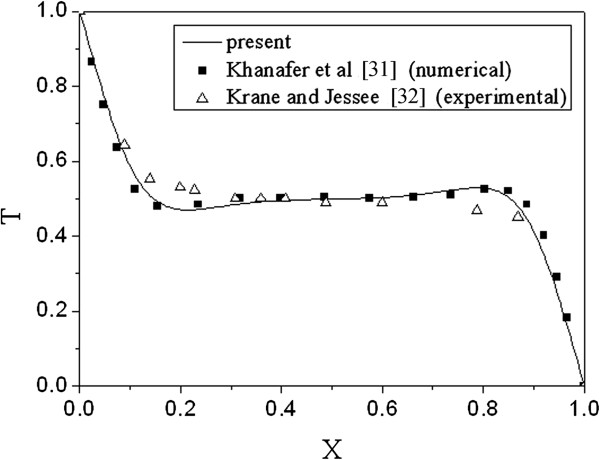
**Temperature distribution at horizontal midsections-sections of the enclosure (*Ra *= 10**^**5**^**, *Pr *= 0.7).**

In order to validate the Lattice Boltzmann model proposed in this work, the temperature distribution at midsections of the enclosure at Ra = 1 × 10^5^, Pr = 0.7 is compared with the numerical results from Khanafer et al. [31] and experimental results from Krane et al. [32] in Figure 2. It can be seen that the results of this paper have a good agreement with those numerical [31] and experimental [32] results. They are closer to the experimental [32] than the numerical [31] results. In addition, the Nusselt number results at different Rayleigh numbers of this paper are compared with other published literature listed in Table 3, and it can be seen that the results are in good agreement.

**Table 3:** **Comparison of average Nusselt numbers with other published data (*Pr *= 0.7)**

	***Ra *= 10**^**3**^	***Ra *= 10**^**4**^	***Ra *= 10**^**5**^	***Ra *= 10**^**6**^
Present work	1.118	2.247	4.522	8.808
D'Orazio et al. [33]	1.117	2.235	4.504	8.767
De Vahl Davis [34]	1.118	2.243	4.519	8.800
Khanafer et al. [31]	1.118	2.245	4.522	8.826

**Figure 3:**
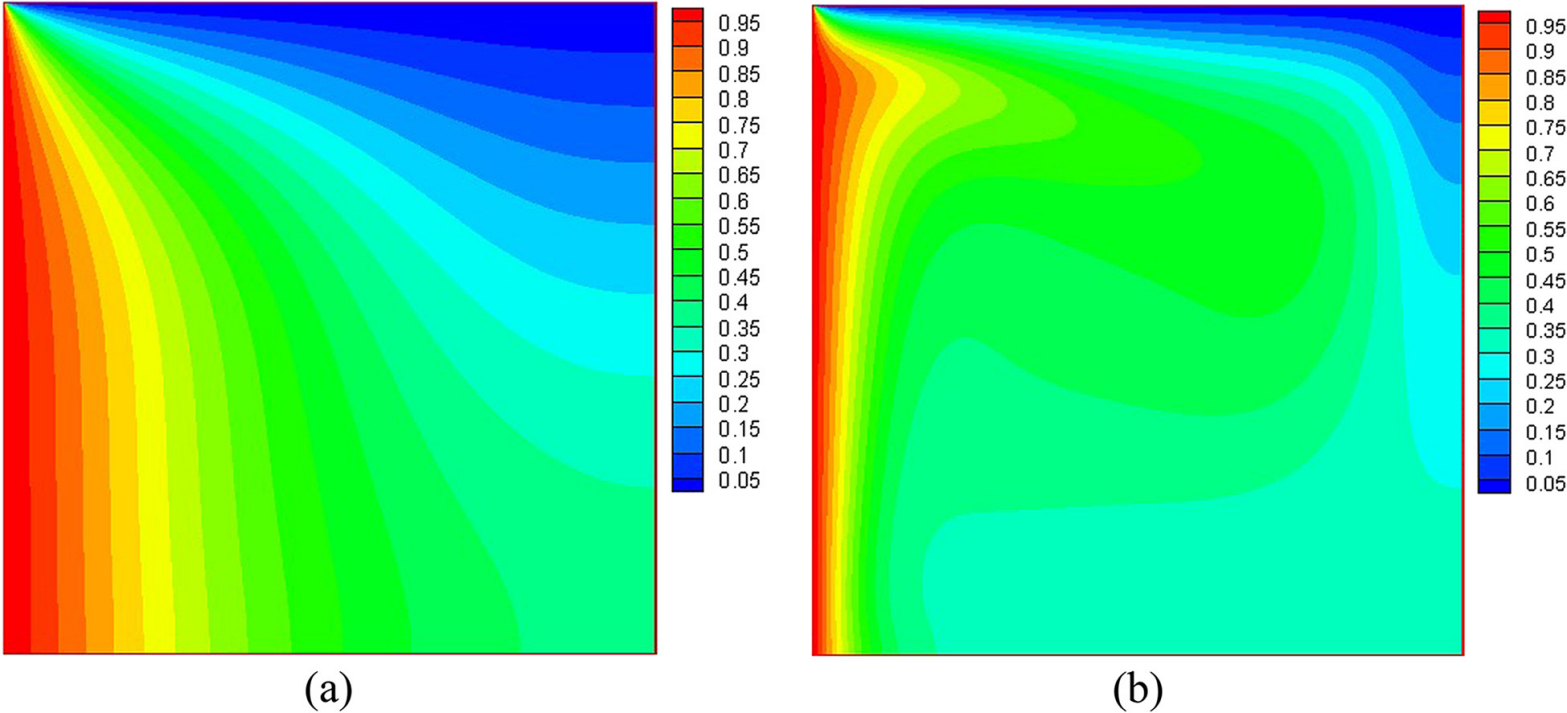
**Temperature nephogram of nanofluid at different Rayleigh numbers (a) *Ra *= 1 × 10**^**3 **^**and (b) *Ra *= 1 × 10**^**5**^**.**

Due to the temperature balance between water and nanoparticles, the temperature nephogram of water and nanoparticles for each of the nanoparticle fractions are identical. The temperature nephograms of nanofluid at Ra = 1 × 10^3^ and Ra = 1 × 10^5^ are presented in Figure 3. It can be seen that isotherms are more crooked with the higher Rayleigh number, which denotes that the heat transfer characteristic transforms from conduction to convection.

**Figure 4:**
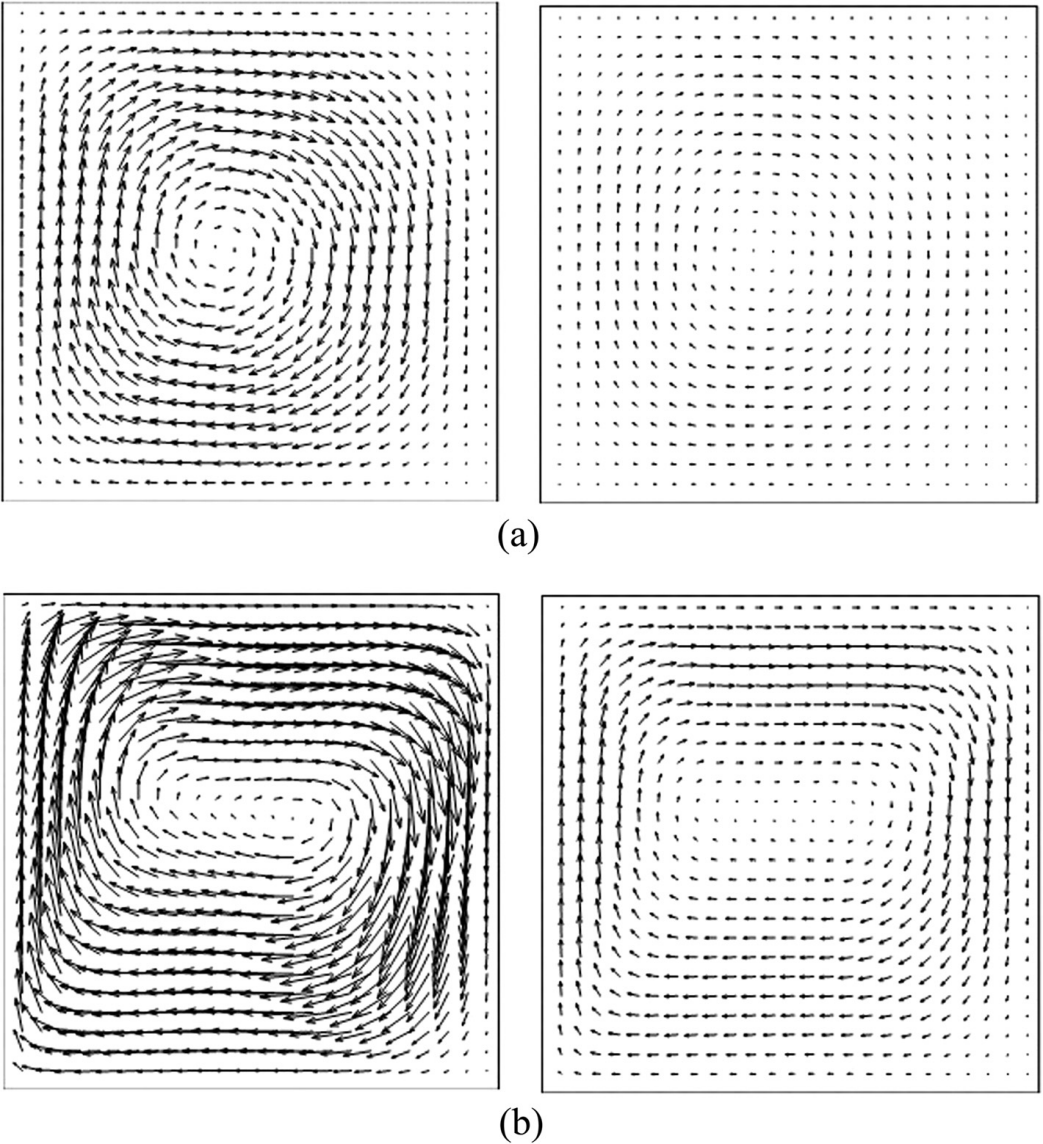
**Velocity vectors of nanofluid components.** Left, water; right, nanoparticles. *φ* = 0.03 **(a)** Ra = 1 × 10^3^, **(b)** Ra = 1 × 10^5^.

Because there are fewer nanoparticles than water molecules, and the drag force of nanoparticles on water is small, the velocity vectors of nanofluid with different nanoparticle fractions have such small differences that it is difficult to distinguish them. However, the differences can be observed in the Nusselt number distribution. For this reason, only the velocity vectors of nanofluid components with *φ* = 0.03 at different Rayleigh numbers are given as an example in Figure 4. Separating the nanofluid into its two constitutive components, it can be seen that the velocity vectors of the water component are larger than those of the nanoparticle component due to the law of conservation of momentum. The velocity difference between the water component and the nanoparticle component gives rise to the drag force. In addition, it can be seen that velocity increases with Rayleigh number, which can also explain that the heat transfer characteristic transforms from conduction to convection.

Driving force and interaction forces have a big effect on nanoparticle volume fraction distribution and the flow and heat transfer characteristics of the nanofluid. The main driving force in this work is the temperature difference. Interaction forces between nanoparticles and base fluid include gravity-buoyancy force, drag force, interaction potential force, and Brownian force. In order to compare the effects of these forces, the ranges of them are presented in Table 4. We used double-precision variables in our code. From Table 4, we can find that the temperature difference driving force ***F***_S_ is much bigger than the other forces (interaction forces between nanoparticles and base fluid). The driving force has the greatest effect on nanoparticle volume fraction distribution, and the effects of other forces on nanoparticle volume fraction distribution can be ignored in this case. However, these other forces play an important role in the flow and heat transfer of the nanofluid. Apart from the temperature difference driving force, the Brownian force is much larger than other forces, which is different from other two-phase fluids. For this reason, the Brownian force can enhance the heat transfer of the nanofluid by disturbing the flow boundary layer and the thermal boundary layer. Drag force comes about due to the velocity difference between nanoparticles and water molecules, and the nanoparticles in the water decrease the velocity of nanofluid in the enclosure, which in turn attenuates the natural convection of nanofluid in the enclosure. The interaction potential force prevents the nanoparticles from gathering together and keeps the nanoparticles dispersed in the water. In addition to the above forces, there is the gravity-buoyancy force, that is, the sum of gravity of the nanoparticles themselves and the buoyancy force of the water. The gravity-buoyancy force and temperature difference driving force together give rise to the velocity vectors of the nanofluid within the enclosure. In summary, Brownian force, interaction potential force, and gravity-buoyancy force contribute to the enhanced natural convective heat transfer, while drag force contributes to the attenuation of heat transfer.

**Table 4:** **Comparison of different forces (*Ra *= 10**^**5**^**, φ = 0.03)**

	**Forces**						
	***F***_**S**_	***F***_**A**_	***F***_**Bx**_	***F***_**By**_	***F***_**H**_	***F***_**Dx**_	***F***_**Dy**_
Minimum	-6E-06	-3.2E-19	-5E-13	2E-14	-9E-19	-8E-16	-1.6E-15
Maximum	6E-06	-2E-20	5E-13	2E-13	-1E-19	1.2E-15	1.6E-15

**Figure 5:**
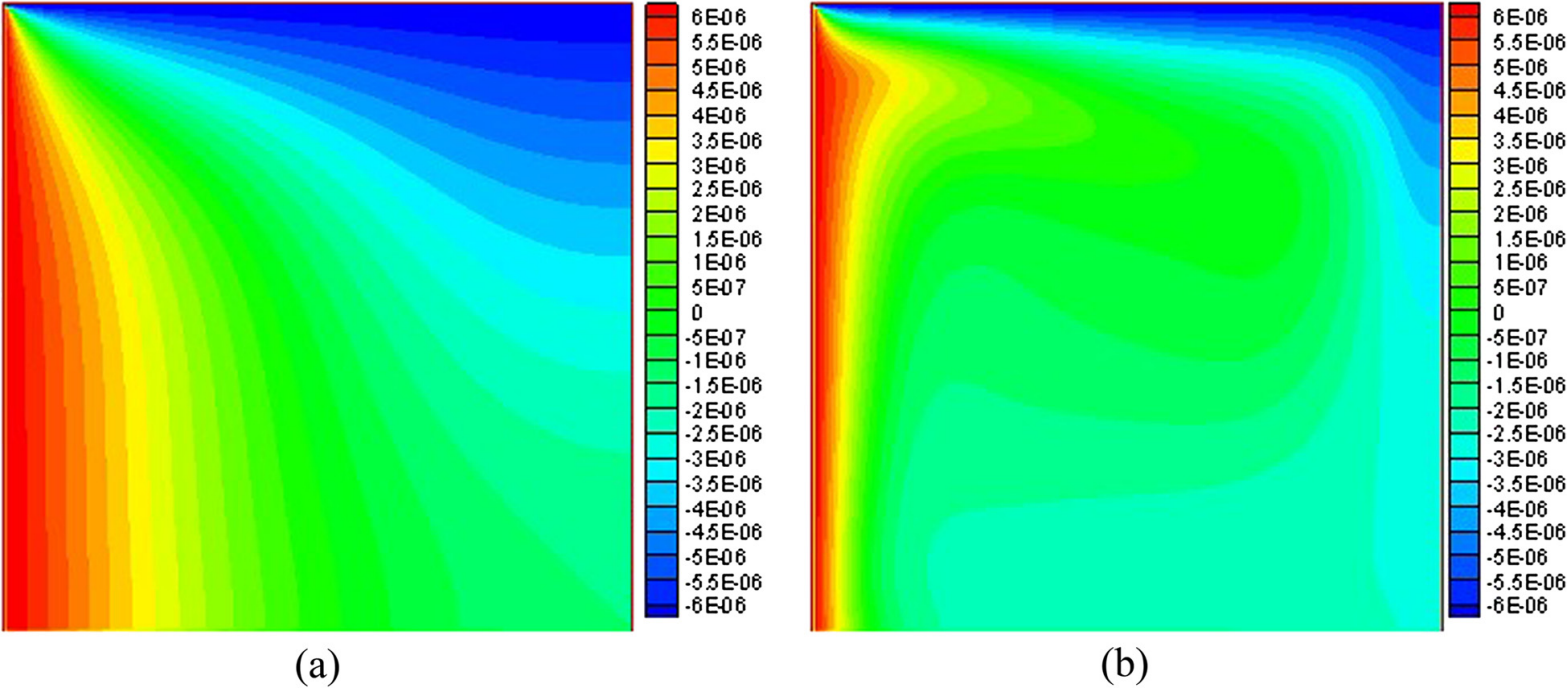
**Temperature difference driving force at different Rayleigh numbers , φ = 0.03 (a) *Ra *= 1 × 10**^**3 **^**(b) *Ra *= 1 × 10**^**5**^**.**

The temperature difference driving force distribution in the square at different Rayleigh numbers is given in Figure 5. From Figure 5, we can see that the temperature difference driving force along the left wall (high temperature) and the top wall (low temperature) is high. Its direction along the high-temperature wall is upward, and that along the low-temperature wall is downward, while the temperature difference driving force in other regions far away from the two walls (left wall and top wall) is small. From Figure 3, it can be seen that the temperature gradient near the left wall and the top wall is higher than that in other regions, which causes a high temperature difference driving force near there. Similarly, the temperature gradient in other regions is small, causing only a low temperature difference driving force in that vicinity. In addition, it can be seen that the same driving force line at a high Rayleigh number becomes more crooked than that at a low Rayleigh number. This is because the driving force is caused by the temperature difference (temperature gradient); a bigger temperature gradient causes the same driving force line to become more crooked. It can be seen from Figure 3 that isotherms are more crooked at a higher Rayleigh number, and the isotherm changes correspond to the changes of temperature gradient. Thus, the conclusion that the same driving force line at a high Rayleigh number becomes more crooked than that that at a low Rayleigh number is obtained.

**Figure 6:**
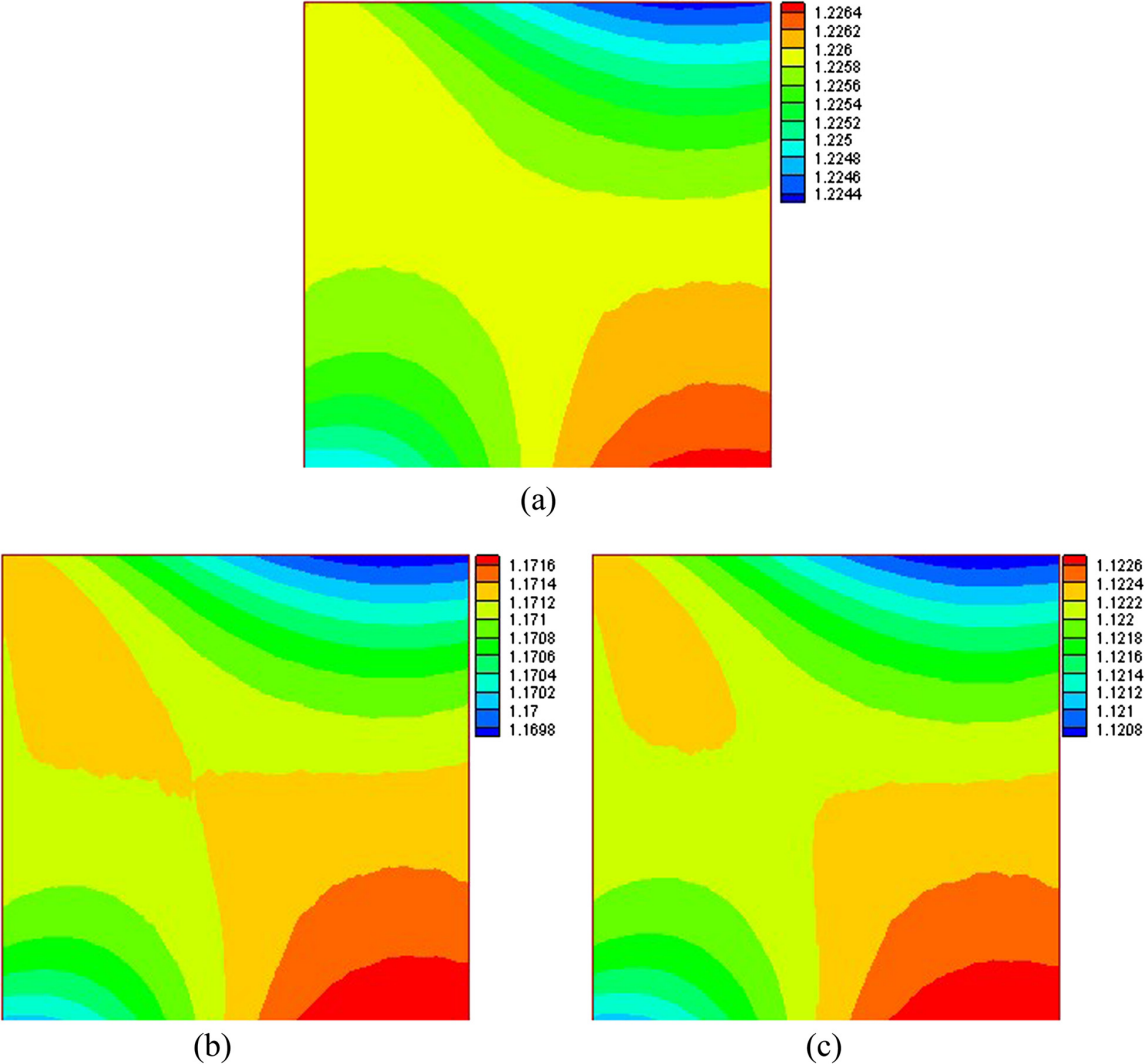
**Density distribution of water phase at *Ra *= 1 × 10**^**3 **^**(a) φ = 0.01 (b) φ = 0.03 (c) φ = 0.05.**

**Figure 7:**
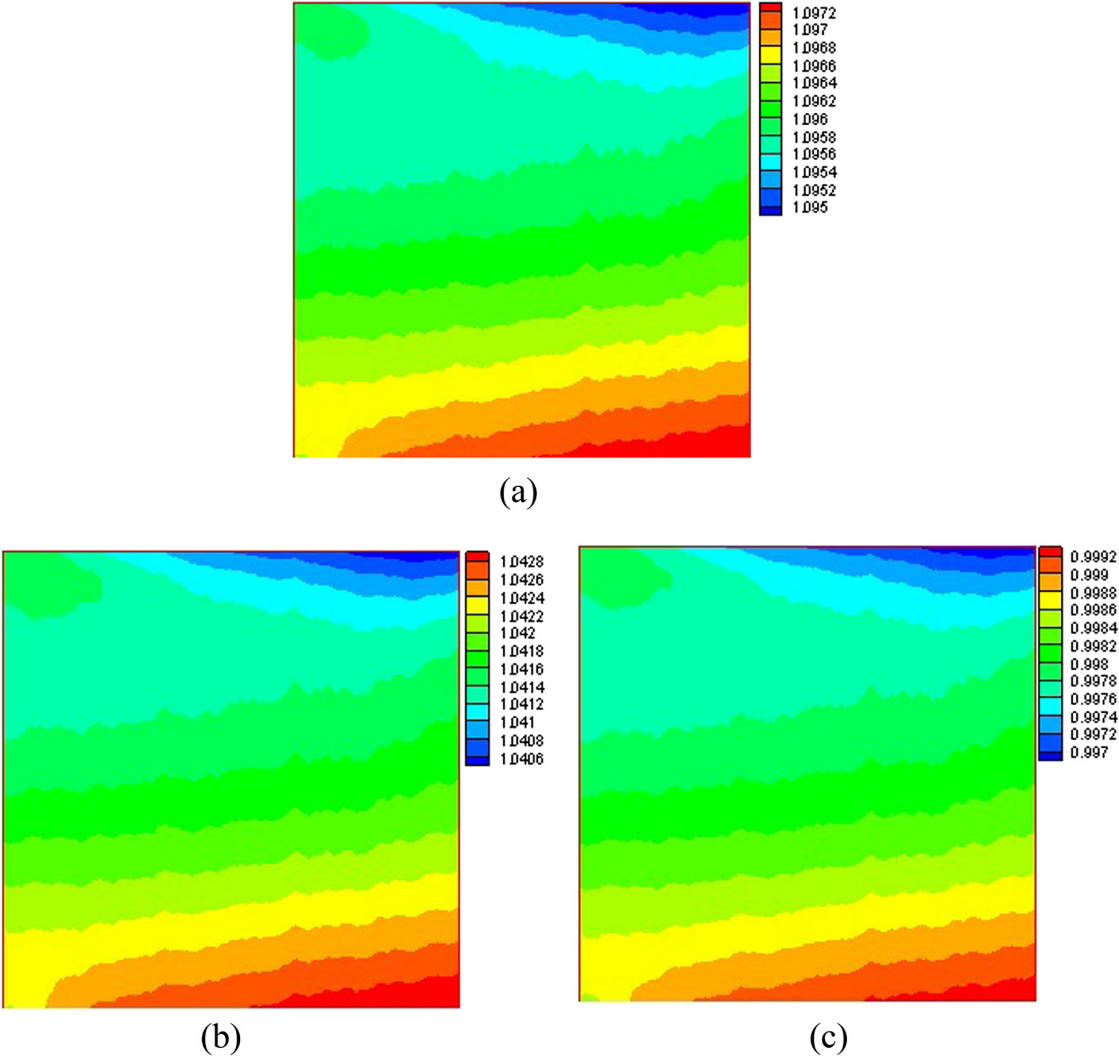
**Density distribution of water phase at *Ra *= 1 × 10**^**5 **^**(a) φ = 0.01 (b) φ = 0.03 (c) φ = 0.05.**

Figures 6 and 7 give the density distribution of the water phase at Ra = 1 × 10^3^ and Ra = 1 × 10^5^. For a low Rayleigh number (Ra = 1 × 10^3^), when the water near the left wall is heated, its density decreases and flows upward, so the density of water near the top right corner also becomes smaller. Then when the water is cooled by the top wall, the density of the water becomes larger. Then the denser water flows downward to the lower right corner, and so, the density of water in the lower right corner is larger than that in other regions. Because the temperature gradient (corresponding to the temperature difference driving force) is small and the temperature is high in the lower left corner, the density of water in the lower left corner is thus low. For a high Rayleigh number (Ra = 1 × 10^5^), the temperature gradient and the corresponding driving force become larger, then the lower-density water, including that in the lower left corner, rises to the top right corner. The denser water is cooled by the top wall and flows downward to the lower right corner, and the area where the denser water in the lower right corner becomes larger.

**Figure 8:**
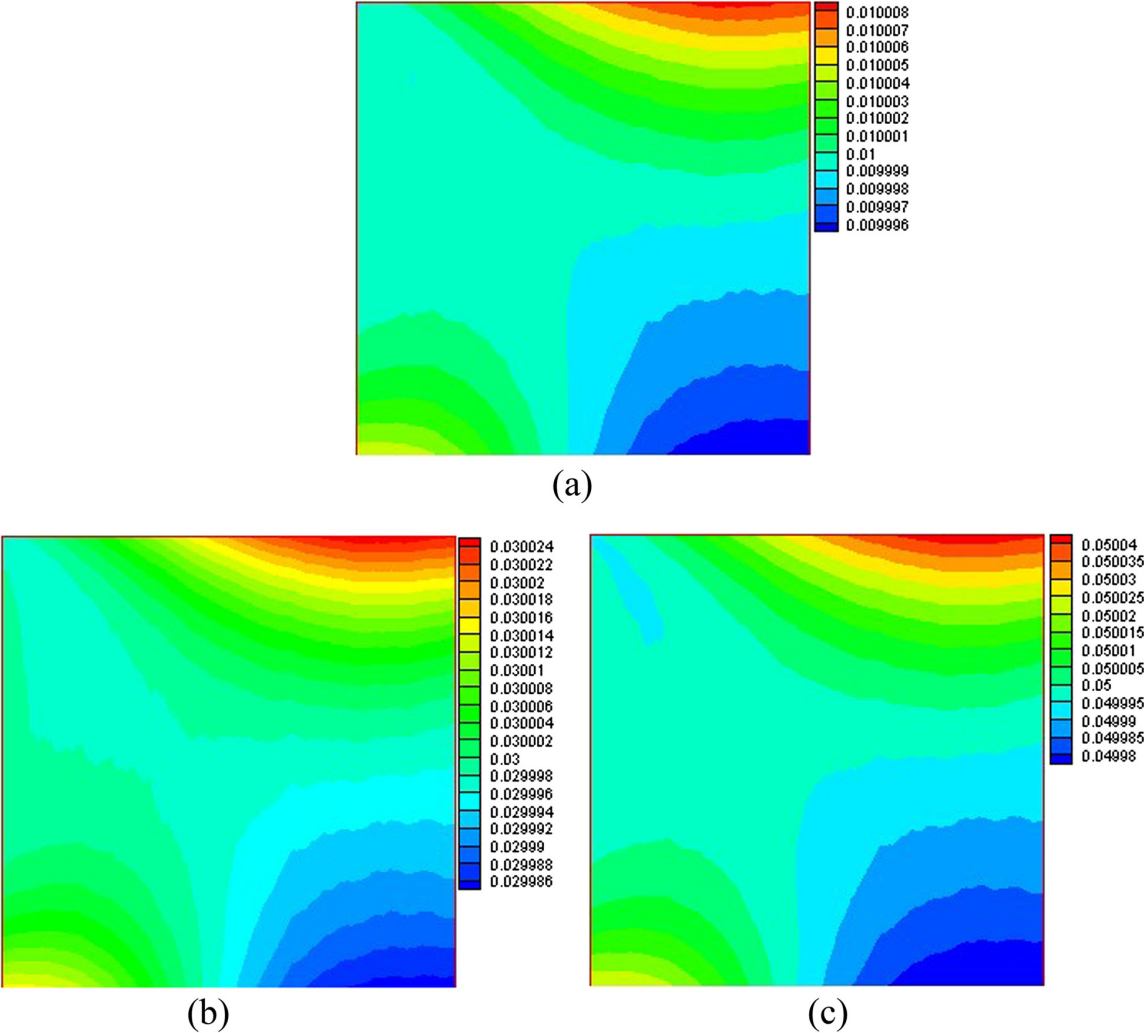
**Nanoparticle volume fraction distribution at Ra = 1 × 10**^**3**^**. (a)***φ* = 0.01, **(b)***φ* = 0.03, and **(c)***φ* = 0.05.

**Figure 9:**
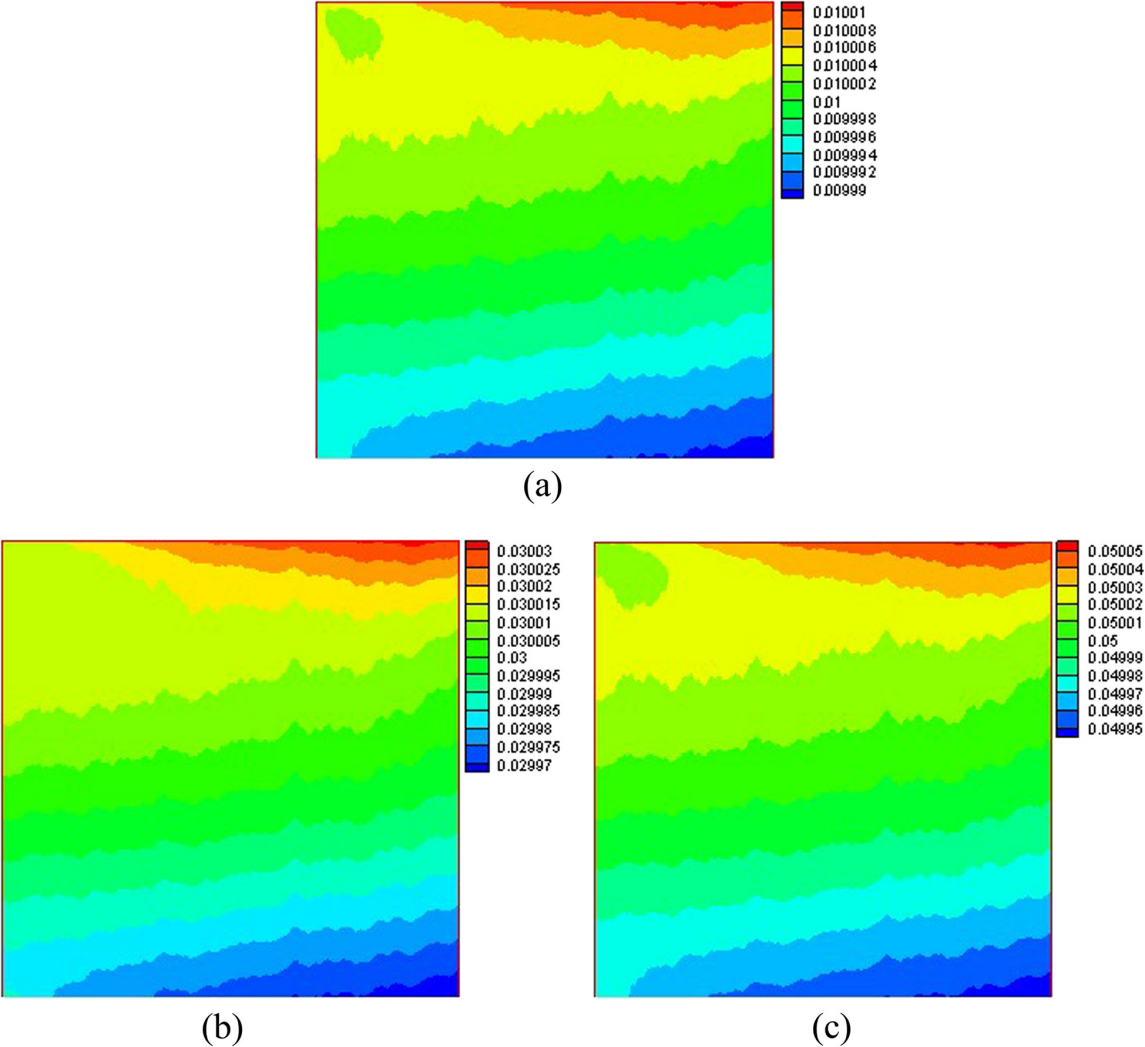
**Nanoparticle volume fraction distribution at Ra = 1 × 10**^**5**^**. (a)***φ* = 0.01, **(b)***φ* = 0.03, and **(c)***φ* = 0.05.

Figures 8 and 9 respectively present the nanoparticle distribution of nanofluid with volume fractions at Ra = 1 × 10^3^ and Ra = 1 × 10^5^. For a low Rayleigh number (Ra = 1 × 10^3^), the driving force along the left wall is upward, and many nanoparticles are driven to the top right corner, which contributes to the high nanoparticle volume fraction in the top right corner. However, the temperature gradient in the lower left corner is small and causes a correspondingly small temperature difference driving force. Thus, many nanoparticles are left in the lower left corner, which contributes to the high nanoparticle volume fraction in the lower left corner. There is a large temperature gradient in the lower right corner, and the large driving force displaces the nanoparticles off the lower right corner, which contributes to the low nanoparticle volume fraction in the lower right corner. For a high Rayleigh number (Ra = 1 × 10^5^), the convection heat transfer is enhanced and the velocity of the nanofluid becomes larger, and the temperature gradient and the corresponding driving force become bigger. Thus, many nanoparticles from the bottom are driven to the top by the driving force, which contributes to the low nanoparticle volume fraction at the bottom and a high nanoparticle volume fraction at the top. In addition, we can see that the nanoparticle volume fraction distribution is opposite to that of the water-phase density distribution. From Table 4, we can see that the temperature difference driving force is the biggest one, and the changes of the water-phase density and the inhomogeneous nanoparticle distribution are mainly due to the driving force. Through the above analysis, it is found that the nanoparticles migrate to locations where the water density is small, and thus, the conclusion that the nanoparticle volume fraction distribution is opposite to that of the water-phase density distribution is obtained.

It is also found that almost all the isolines behave with oscillations in Figures 6, 7, 8, 9, but smooth isolines are given in Figures 3 and 5. Due to the ruleless Brownian movement of nanoparticles, it is difficult for nanofluid to achieve a complete equilibrium state, which is the difference compared with other common two-phase fluids. In order to expediently judge the equilibrium state and save time, we choose the temperature equilibrium states of water phase and nanoparticle phase as the whole nanofluid equilibrium state in the computation. When the water-phase and nanoparticle-phase temperatures all achieve equilibrium state, the whole nanofluid (temperature distribution, velocity vectors, density distribution, and nanoparticle volume fraction distribution) is considered as being in an equilibrium state. Hence, the temperature isolines in Figures 3 and 5 look smooth due to a complete equilibrium state, and the density distribution in Figures 6 and 7 and nanoparticle volume fraction distribution in Figures 8 and 9 behave with oscillations due to an approximate equilibrium state. Although the interparticle interaction forces have little effect on heat transfer, they play an important role on the nanoparticle distribution.

**Figure 10:**
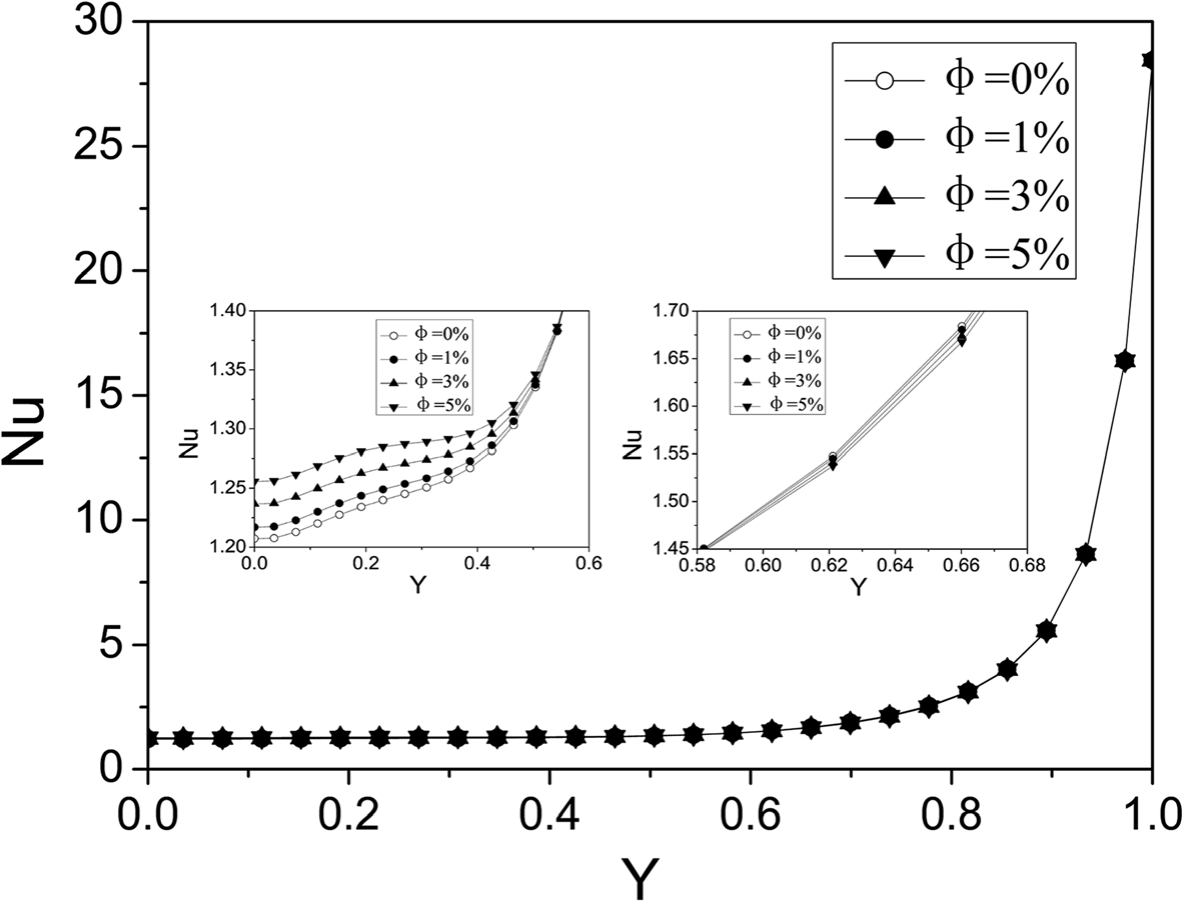
**Nusselt number distribution along the heated surface using Al**_**2**_**O**_**3**_**-water nanofluid at *Ra *= 10**^**3**^**.**

Figure 10 shows the Nusselt number distribution along the heated surface using Al_2_O_3_-water nanofluid at Ra = 10^3^. It can be seen that the Nusselt number along the heated surface increases with nanoparticle volume fraction at low Y (0 < Y < 0.58) and decreases with nanoparticle volume fraction at high Y (0.58 < Y < 1). Because the heat transfer is more sensitive to thermal conductivity than viscosity at low Y, while it is more sensitive to viscosity than thermal conductivity at high Y.

**Figure 11:**
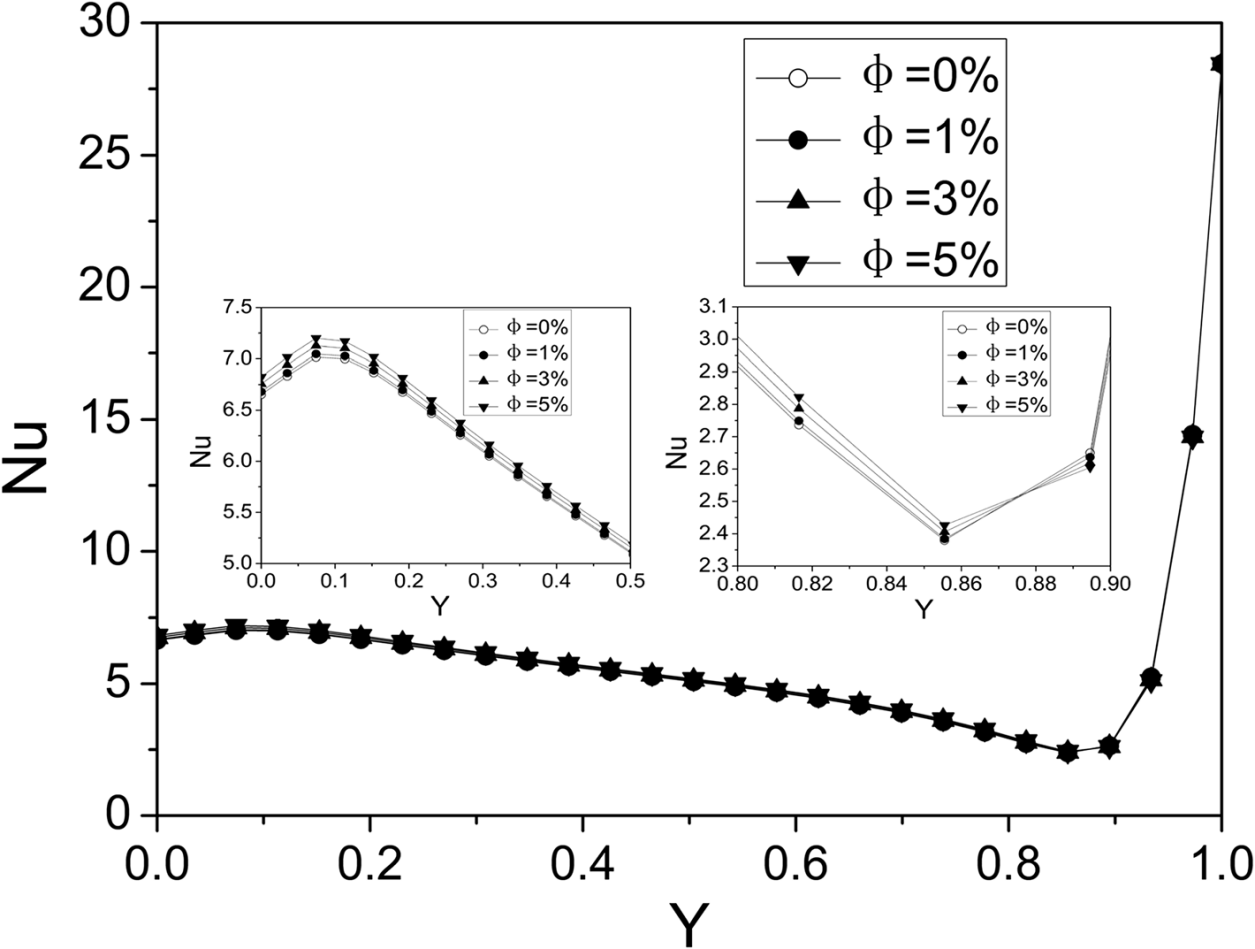
**Nusselt number distribution along the heated surface using Al**_**2**_**O**_**3**_**-water nanofluid at *Ra *= 10**^**5**^**.**

Figure 11 shows Nusselt number distribution along the heated surface using Al_2_O_3_-water nanofluid at *Ra* = 10^5^. It can be seen that the Nusselt number along the heated surface increases with nanoparticle volume fraction at low Y (0 < Y < 0.875) and decreases with nanoparticle volume fraction at high Y (0.875 < Y < 1). Compared with Figure 7, the Nusselt number becomes larger, and the enhanced heat transfer section also gets longer. The high Rayleigh number increases the velocity and then enhances the heat transfer.

**Figure 12:**
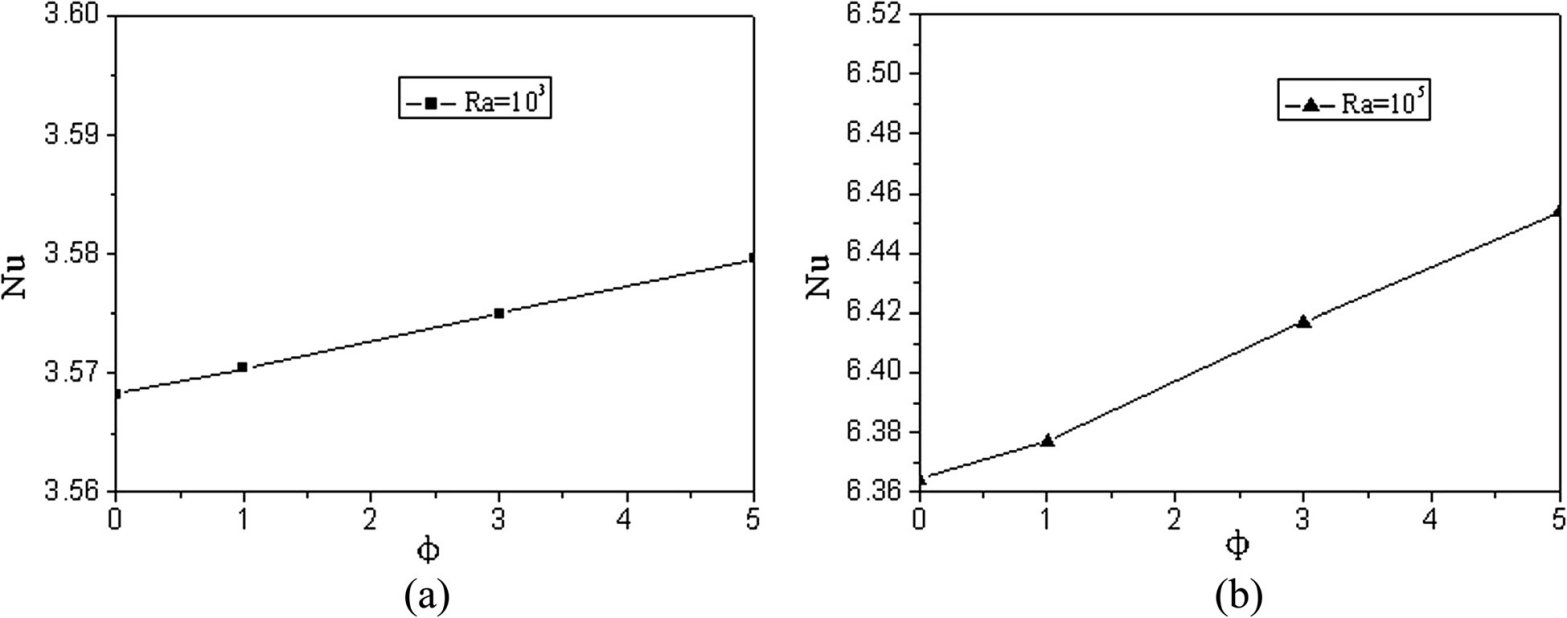
**Average Nusselt numbers at different Rayleigh numbers (a) *Ra *= 10**^**3 **^**and (b) *Ra *= 10**^**5**^**.**

Figure 12 presents the average Nusselt numbers at different Rayleigh numbers. Although the Nusselt number distribution along the heated surface increases with nanoparticle volume fraction in one section and decreases in the other section, the average Nusselt numbers at *Ra* = 10^3^ and *Ra* = 10^5^ both increase with nanoparticle volume fraction. For this square enclosure (left wall is kept at a high constant temperature (*T*_H_), and top cold wall is kept at a low constant temperature (*T*_C_)), adding nanoparticles can enhance the average heat transfer at both a low and a high Rayleigh number. In addition, the enhancement of the average Nusselt numbers is much more pronounced at a high Rayleigh number than at a low Rayleigh number.

## Conclusion

A 2D two phase Lattice Boltzmann model has been developed for nanofluids and the simulation results of this two-phase Lattice Boltzmann model are in good agreement with published experimental results. This model is applied to investigate the natural convection of a square enclosure filled with Al_2_O_3_ nanofluid. The effects of different nanoparticle fractions and Rayleigh numbers on natural convection heat transfer of nanofluid are investigated. In addition the effects of forces on the nanoparticles volume fraction distribution and the heat transfer are also investigated.

It is found that the Nusselt number distribution along the heated surface firstly increases, and then decreases with Y at both low and high Rayleigh numbers. Average Nusselt numbers of the whole square enclosure both increase with nanoparticles volume fraction at a low and a high Rayleigh number. In addition, the enhancement of the average Nusselt numbers is much more pronounced at a high Rayleigh number than at a low Rayleigh number.

It is found that the temperature difference driving force is the biggest force and has the greatest effect on nanoparticle volume fraction distribution. For a low Rayleigh number, the nanoparticle volume fraction is low in the lower right corner and high in the top right corner and lower left corner. For a high Rayleigh number, the nanoparticle volume fraction is low at the bottom and high at the top.

Apart from the temperature difference driving force, Brownian force, interaction potential force, and gravity-buoyancy force contribute to the enhanced natural convective heat transfer, while the drag force contributes to the attenuation of heat transfer.

## Nomenclature

*a* radius of nanoparticle (m)

*A* Hamaker constant

*B*_*a*_ weight coefficient

*c* reference lattice velocity

*c*_s_ lattice sound velocity

*c*_p_ specific heat capacity (J/kg K)

*e*_*α*_ lattice velocity vector

$$f_{\alpha }^{\sigma }$$

density distribution function

$$f_{\alpha }^{\mathrm{\sigma eq}}$$

local equilibrium density distribution function

$$F_{\alpha }^{\sigma '}$$

dimensionless external force in direction of lattice velocity

$$F_{\alpha }^{\sigma }$$

dimensionless total interparticle interaction forces

*F*_S_ dimensionless temperature difference driving forces

*F*_B_ dimensionless Brownian force

*F*_H_ dimensionless gravity and buoyancy force

*F*_D_ dimensionless drag force

*F*_A_ dimensionless interaction potential force

*g* dimensionless gravitational acceleration

*G* dimensionless effective external force

*G*_*i*_ Gaussian random number

*h*_*aβ*_ convective heat transfer coefficient (W/(m^2^ K))

*H* dimensionless characteristic length of the square cavity

*k* thermal conductivity coefficient (W/m/K)

*k*_*B*_ Boltzmann constant

*L*_cc_ center-to-center distance between particles (m)

Ma Mach number

*m*^*σ*^ mass of a single nanoparticle (kg)

*n*_*i*_ number of the particles within the adjacent lattice *i*

Nu Nusselt number

Pr Prandtl number

*r* position vector

Ra Rayleigh number

*t* time (s)

$$T_{\alpha }^{\sigma }$$

temperature distribution function

$$T_{\alpha }^{\mathrm{\sigma eq}}$$

local equilibrium temperature distribution function

*T* dimensionless temperature

*T*_0_ dimensionless average temperature (*T*_0_ = (*T*_H_ + *T*_C_)/2)

*T*_H_ dimensionless hot temperature

*T*_C_ dimensionless cold temperature

*u*^*σ*^ dimensionless macro-velocity

*u*_c_ dimensionless characteristic velocity of natural convection

*V*_*A*_ dimensionless interaction potential

*V* volume of a single lattice (m^3^)

*w*_α_ weight coefficient

*x*, *y* dimensionless coordinates

## Greek symbols

*β*^*σ*^ thermal expansion coefficient (K^-1^)

*ρ*^*σ*^ density (kg/m^3^)

*v* kinematic viscosity (m^2^/s)

*η* dynamic viscosity (Pa s)

*χ* thermal diffusion coefficient (m^2^/s)

*γ* surface tension (N/m)

*φ* nanoparticle volume fraction

*δ*_*x*_ lattice step

*δ*_*t*_ time step

*σ* components (*σ* = 1, 2, water and nanoparticles)

*τ*_*f*_ dimensionless collision-relaxation time for the flow field

*τ*_*T*_ dimensionless collision-relaxation time for the temperature field

*∆T* dimensionless temperature difference (*∆T* = *T*_*H*_ – *T*_*C*_)

*Δρ*^’^ dimensionless mass density difference between nanoparticles and base fluid

*∆u* dimensionless velocity difference between nanoparticles and base fluid

*Φ*_*αβ*_ dimensionless energy exchange between nanoparticles and base fluid

Error_1_ maximal relative error of velocities between two adjacent time layers

Error_2_ maximal relative error of temperatures between two adjacent time layers

## Subscripts

*α* lattice velocity direction

avg average

C cold

nf nanofluid

H hot

w base fluid

p nanoparticle

## Competing interests

The authors declare that they have no competing interests.

## Authors' contributions

CQ participated in the design of the program, carried out the numerical simulation of nanofluid, and drafted the manuscript. YRH conceived of the study, participated in the design of the program, and checked the grammar of the manuscript. SNY, FLT, and YWH participated in the design of the program. All authors read and approved the final manuscript.

## References

[B1] Wang SX Zhou Y Guan W Ding B Preparation and characterization of stimuli-responsive magnetic nanoparticles Nanoscale Res Lett 2008 3 289 294 10.1007/s11671-008-9151-3

[B2] Bora DK Deb P Fatty acid binding domain mediated conjugation of ultrafine magnetic nanoparticles with albumin protein Nanoscale Res Lett 2009 4 138 143 10.1007/s11671-008-9213-6 20596435 PMC2893867

[B3] Guo SZ Li Y Jiang JS Xie HQ Nanofluids containing γ-Fe_2_O_3_ nanoparticles and their heat transfer enhancements Nanoscale Res Lett 2010 5 1222 1227 10.1007/s11671-010-9630-1 20596461 PMC2893802

[B4] Pinilla MG Martínez E Vidaurri GS Tijerina EP Deposition of size-selected Cu nanoparticles by inert gas condensation Nanoscale Res Lett 2010 5 180 188 10.1007/s11671-009-9462-z PMC2893918 20652132

[B5] Yang X Liu Z A kind of nanofluid consisting of surface-functionalized nanoparticles Nanoscale Res Lett 2010 5 1324 1328 10.1007/s11671-010-9646-6 20676194 PMC2897029

[B6] Zhu H Han D Meng Z Wu D Zhang C Preparation and thermal conductivity of CuO nanofluid via a wet chemical method Nanoscale Res Lett 2011 6 1 6 21711693 10.1186/1556-276X-6-181 PMC3211235

[B7] Nadeem S Lee C Boundary layer flow of nanofluid over an exponentially stretching surface Nanoscale Res Lett 2012 7 1 6 10.1186/1556-276X-7-1 22289390 PMC3305502

[B8] Wang L Fan J Nanofluids research: key issues Nanoscale Res Lett 2010 5 1241 1252 10.1007/s11671-010-9638-6 20676214 PMC2898525

[B9] Oztop HF Abu-Nada E Numerical study of natural convection in partially heated rectangular enclosures filled with nanofluids Int J Heat Fluid Flow 2008 29 1326 1336 10.1016/j.ijheatfluidflow.2008.04.009

[B10] Ho CJ Chen MW Li ZW Numerical simulation of natural convection of nanofluid in a square enclosure: effects due to uncertainties of viscosity and thermal conductivity Int J Heat Mass Transfer 2008 51 4506 4516 10.1016/j.ijheatmasstransfer.2007.12.019

[B11] Saleh H Roslan R Hashim I Natural convection heat transfer in a nanofluid-filled trapezoidal enclosure Int J Heat Mass Transfer 2011 54 194 201 10.1016/j.ijheatmasstransfer.2010.09.053

[B12] Ghasemi B Aminossadati SM Brownian motion of nanoparticles in a triangular enclosure with natural convection Int J Therm Sci 2010 49 931 940 10.1016/j.ijthermalsci.2009.12.017

[B13] Santra AK Sen S Chakraborty N Study of heat transfer augmentation in a differentially heated square cavity using copper–water nanofluid Int J Therm Sci 2008 47 1113 1122 10.1016/j.ijthermalsci.2007.10.005

[B14] Aminossadati SM Ghasemi B Natural convection cooling of a localised heat source at the bottom of a nanofluid filled enclosure Eur J Mech B/Fluid 2009 28 630 640 10.1016/j.euromechflu.2009.05.006

[B15] Kargar A Ghasemi B Aminossadati SM An artificial neural network approach to cooling analysis of electronic components in enclosures filled with nanofluids J Electron Packaging 2011 133 1 9

[B16] Abu-Nada E Chamkha AJ Effect of nanofluid variable properties on natural convection in enclosures filled with a CuO-EG-water nanofluid Int J Therm Sci 2010 49 2339 2352 10.1016/j.ijthermalsci.2010.07.006

[B17] Hwang KS Lee JH Jang SP Buoyancy-driven heat transfer of water-based Al_2_O_3_ nanofluids in a rectangular cavity Int J Heat Mass Transfer 2007 50 4003 4010 10.1016/j.ijheatmasstransfer.2007.01.037

[B18] Jang SP Choi SUS Role of Brownian motion in the enhanced thermal conductivity of nanofluids Appl Phys Lett 2004 84 4316 4318 10.1063/1.1756684

[B19] Barrios G Rechtman R Rojas J Tovar R The lattice Boltzmann equation for natural convection in a two-dimensional cavity with a partially heated wall J Fluid Mech 2005 522 91 100

[B20] Peng Y Shu C Chew YT Simplified thermal lattice Boltzmann model for incompressible thermal flows Phys Rev E 2003 68 026701 10.1103/PhysRevE.68.026701 14525142

[B21] He X Chen S Doolen GD A novel thermal model for the lattice Boltzmann method in incompressible limit J Comput Phys 1998 146 282 300 10.1006/jcph.1998.6057

[B22] Nemati H Farhadi M Sedighi K Fattahi E Darzi AAR Lattice boltzmann simulation of nanofluid in lid-driven cavity Int Commun Heat Mass Transfer 2010 37 1528 1534 10.1016/j.icheatmasstransfer.2010.08.004

[B23] Wang J Wang M Li Z A lattice Boltzmann algorithm for fluid–solid conjugate heat transfer Int J Therm Sci 2007 46 228 234 10.1016/j.ijthermalsci.2006.04.012

[B24] Dixit HN Babu V Simulation of high Rayleigh number natural convection in a square cavity using the lattice Boltzmann method Int J Heat Mass Transfer 2006 49 727 739 10.1016/j.ijheatmasstransfer.2005.07.046

[B25] Peng Y Shu C Chew YT A 3D incompressible thermal lattice Boltzmann model and its application to simulate natural convection in a cubic cavity J Comput Phys 2003 193 260 274

[B26] Xuan Y Yao Z Lattice Boltzmann model for nanofluids Heat Mass Transfer 2005 41 199 205

[B27] Russel WB Saville DA Schowalter WR Colloidal Dispersion 1989 Cambridge: Cambridge University Press

[B28] He C Ahmadi G Particle deposition in a nearly developed turbulent duct flow with electrophoresis J Aerosol Sci 1999 30 739 758 10.1016/S0021-8502(98)00760-5

[B29] Abu-Nada E Effects of variable viscosity and thermal conductivity of Al_2_O_3_-water nanofluid on heat transfer enhancement in natural convection Int J Heat Fluid Flow 2009 30 679 690 10.1016/j.ijheatfluidflow.2009.02.003

[B30] Hortmann M Peric M Scheuerer G Finite volume multigrid prediction of laminar natural convection: benchmark solutions Int J Numer Methods Fluid 1990 11 189 207 10.1002/fld.1650110206

[B31] Khanafer K Vafai K Lightstone M Buoyancy-driven heat transfer enhancement in a two-dimensional enclosure utilizing nanofluids Int J Heat Mass Transfer 2003 46 3639 3653 10.1016/S0017-9310(03)00156-X

[B32] Krane RJ Jessee J Some detailed field measurements for a natural convection flow in a vertical square enclosure Proc 1st ASME-JSME Thermal Eng Joint Conf 1983 1 323 329

[B33] D'Orazio A Corcione M Celata GP Application to natural convection enclosed flows of a lattice Boltzmann BGK model coupled with a general purpose thermal boundary condition Int J Therm Sci 2004 43 575 586 10.1016/j.ijthermalsci.2003.11.002

[B34] De Vahl DG Natural convection of air in a square cavity: a bench mark numerical solution Int J Numer Meth Fluids 1983 3 249 264 10.1002/fld.1650030305

